# Innovation and Access to Medicines for Neglected Populations: Could a Treaty Address a Broken Pharmaceutical R&D System?

**DOI:** 10.1371/journal.pmed.1001218

**Published:** 2012-05-15

**Authors:** Suerie Moon, Jorge Bermudez, Ellen 't Hoen

**Affiliations:** 1 Forum on Global Governance for Health, Harvard Global Health Institute, Cambridge, Massachusetts, United States of America; 2 Harvard School of Public Health, Boston, Massachusetts, United States of America; 3 Fundação Oswaldo Cruz, Rio de Janeiro, Brazil; 4 IS Academy HIV/AIDS, School for Social Science Research, University of Amsterdam, The Netherlands

## Abstract

As part of a cluster of articles leading up to the 2012 World Health Report and critically reflecting on the theme of “no health without research,” Suerie Moon and colleagues argue for a global health R&D treaty to improve innovation in new medicines and strengthening affordability, sustainable financing, efficiency in innovation, and equitable health-centered governance.

**Summary Points**

- The current system for the research and development (R&D) of new medicines does not adequately meet the needs of the majority of the world's population.
- There is a lack of new medicines for the “neglected diseases”—those that primarily affect populations with little purchasing power, and therefore offer an insufficient incentive for industry to invest in R&D. However, with problems extending far beyond the narrow notion of neglected diseases, the issue is better understood as one of “neglected populations.”
- International debate and proposals for reform have ensued, including the recommendation that governments begin negotiations over a binding medical R&D convention to address systematic, long-standing problems with innovation and globally equitable access to medicines. Despite the emergence of many new approaches to generating R&D that meets the needs of poorer populations, efforts remain ad hoc, fragmented, and insufficient.
- We discuss how an R&D treaty could complement and build on existing initiatives by addressing four areas where the system remains particularly weak: affordability, sustainable financing, efficiency in innovation, and equitable health-centered governance.
- We argue that effective tools for global governance are required to generate medical R&D as a global public good, based on the understanding that a politically and financially sustainable system will require both fair contributions from all, and fair benefit-sharing for all.

*In anticipation of the 2012* World Health Report, *this paper was commissioned to help contextualize and critically reflect on the theme of “no health without research.”*

## Introduction

Over the last two decades, recognition has grown that the current system for the research and development (R&D) of new medicines does not adequately meet the needs of the majority of the world's population [1]–[4], over 80% of which lives in low- and middle-income countries (LMICs) [5]. (We use the general term “medicines” to refer broadly to drugs, vaccines, diagnostics, and other medical products.)

The clearest illustration of these shortcomings is the lack of new medicines for the “neglected diseases”—those that primarily affect populations with little purchasing power, and therefore offer an insufficient incentive for industry to invest in R&D. However, the problems with the existing system extend far beyond the narrow notion of neglected diseases. The challenge is better understood as one of “neglected populations”—that is, of ensuring that the global R&D system meets the needs of all, especially of the poorest and most vulnerable populations. Such needs include not only new treatments for neglected diseases, but also access to antimicrobials, affordable medicines for diseases with global incidence such as diabetes and cancer, and products well-adapted for use in resource-limited settings. Thus far, the existing system has largely failed to deliver on these objectives.

These problems have prompted extensive international debate and proposals for reform. After a 2-year intergovernmental negotiation, in 2008 governments agreed upon the World Health Organization (WHO) Global Strategy and Plan of Action on Public Health, Innovation and Intellectual Property, which recognized that the current system fell short in meeting the needs of developing countries with respect to both communicable and non-communicable diseases [4]. In April 2012, the WHO Consultative Expert Working Group on R&D: Financing and Coordination (CEWG) recommended that governments begin negotiations over a global medical R&D convention to address some of these problems in a systematic way [6] (see Box 1). Why might a binding international convention be needed?

**Box 1. A Binding WHO Convention on R&D**

The idea of a binding international convention on R&D has been debated since at least 2004, after an initial proposal by Hubbard and Love [55],[60]. Over the years, it has received support from a number of governments, scientists, Nobel Laureates, civil society organizations, and other experts [56]–[58],[61]–[63]. Key features of treaty proposals include binding obligations on governments to invest in R&D, equitable distribution of contributions across countries, priorities driven by health needs, measures to ensure affordability of the end product, and innovative approaches to incentivizing R&D [55],[56],[58]. Such a treaty could be crafted under the auspices of the WHO, whose Constitution allows for its 194 member states to negotiate formal international law (such as treaties, agreements, or conventions). Article 19 of the Constitution requires approval from two-thirds of member states to adopt any such agreement, and also allows individual member states to choose not to sign onto the agreement [64]. While both formal and informal norms (such as guidelines or global strategies) can influence the behavior of states and non-state actors, binding international law offers several potential advantages: it can provide for more predictable, reliable, and stable norms; carry stronger normative force, even in the absence of enforcement mechanisms; and can be more enforceable at national level [65]. An important precedent was set with the 2005 Framework Convention on Tobacco Control, the first public health treaty negotiated within WHO, which has contributed significantly to global tobacco control efforts [63],[66].

## Shortcomings of the Current R&D System

Today, patents are the main policy tool to drive investments into medicines R&D (Box 2) Prior to the 1990s, there was great variation among countries in the types and length of patents available—on average, industrialized countries granted longer patent terms (15–17 years), developing countries granted shorter terms (5–10 years), and many countries—including Western European nations—made special exceptions for food, medicines, and agricultural technologies in their national patent laws [7],[8]. However, since the 1995 entry into force of the World Trade Organization (WTO) Agreement on Trade-Related Aspects of Intellectual Property Rights (TRIPS), countries were required to harmonize their patent laws to the level of those developed in Western countries with large pharmaceutical industries. As a result, medicines are now subject to minimum 20-year patent terms in most WTO Members (except in least-developed country members, which have an extension until at least 2016 [9]).

**Box 2. The Current Global R&D System**

Total public and private global investment in pharmaceutical R&D was estimated at US$160 billion in 2005, of which about 97% came from high-income countries and 51% from the pharmaceutical industry [50],[67]. In brief, the current R&D system works as follows: governments invest in early-stage basic research, which is often carried out in public laboratories or academic institutions. The pharmaceutical industry then takes up promising leads and invests further in the development of a product, carrying out clinical trials to test if a medicine is safe and efficacious, then filing for regulatory approval. If successful, firms then market, sell, and distribute the medicine, usually under the protection provided by one or more patents and other regulatory measures; the higher prices enabled by these patents allow firms to recoup their R&D investments and are paid by consumers or by public or private health insurance.

American economic historian Paul A. David has likened the patent system to a “panda's thumb”—a product of centuries of evolution but poorly suited as a policy tool for modern innovation [10]. Why was this system globalized?

Medical knowledge has the potential to be a global public good—that is, knowledge produced in one country can benefit the entire global community (it is “non-excludable”), and disclosing that knowledge does not reduce the amount of knowledge left for others to enjoy (it is “non-rival”) [11]. While there are potentially great social benefits from the public goods nature of medical knowledge, it also raises the question of how the burden of paying for such knowledge should be distributed globally. If one country can benefit from the investment of another, there is a powerful temptation to “free-ride” on the other's efforts; the end result may be aggregate global underinvestment in R&D. One rationale for the global harmonization of patent policies was to distribute the burden of financing R&D more uniformly across more countries, and to prevent free-riding [12],[13].

A key problem, however, was the application of uniform patent rules in a world with great wealth disparities: the average per capita income across all high-income countries (US$37,719) was 30 times that across all low- and lower-middle income countries (US$1,270) [14]. Yet, a patent allows a company to price a medicine at the same level in the United States as in India, as some firms have chosen to do [15],[16]. While a globalized patent system may be effective in dealing with the free rider problem, it does not equitably distribute the costs of R&D and can block access to medicines for a large proportion of the population.

Growing dissatisfaction with the existing system has raised the following question: could there be a more politically sustainable way to distribute R&D costs across countries so that equitable access to medicines is not sacrificed?

## New Approaches to Access and Innovation

Various policy experiments have been implemented or proposed to address both the access and innovation problems outlined above. HIV/AIDS provides the most significant example of improved access to widely patented medicines. Access to antiretroviral (ARV) medicines for HIV has increased over 14-fold since 2003, to reach 6.6 million people in 2010 [17]; such progress was enabled, in part, by low-cost generic ARVs made widely available through the use of TRIPS flexibilities by governments, the policies of patent holders, and the availability of international funding to support treatment programs [18].

In the area of innovation, there has been increased funding for specific neglected diseases [19], technology transfer to build research and production capacity in developing countries [20],[21], market creation to induce the development of pediatric formulations of ARVs [22], an advanced market commitment to generate adapted pneumococcal vaccines [23], a priority regulatory review voucher granted in exchange for bringing a neglected disease drug to market [24], the implementation of smaller milestone prizes and proposals for larger end-product prizes [25]–[32], upstream and downstream patent pools [33]–[35], and open-source approaches [36]–[38].

Of particular note are the product development partnerships (PDPs) dedicated to neglected disease R&D, largely financed through public and philanthropic funds [19],[39],[40]. Two examples of PDPs' potential to develop drugs at relatively low-cost are the Drugs for Neglected Diseases initiative (DNDi) and Medicines for Malaria Venture (MMV). From 2003 to 2011, with an investment of EUR100 million, DNDi built a pipeline of potential new drugs and developed six new products, including five combination treatments for malaria, sleeping sickness, and visceral leishmaniasis and a pediatric formulation for Chagas disease [41]. Over a 10-year period with a budget of US$310 million, MMV developed three new products, including a pediatric formulation, injectable artesunate, and a new fixed-dose combination, and built a pipeline of nearly 60 projects [42]. The costs of a non-profit PDP model that relies on contributions from and cooperation with both public and private partners cannot be compared directly to the pharmaceutical industry. Yet, the difference in scale between PDP and industry costs is striking, and underscores the need to give alternate models serious consideration. One estimate pegged the average private-sector cost to develop a new drug at US$1.3 billion [43], though this figure has been contested and remains controversial [44],[45]. Indeed, the pharmaceutical industry itself is testing new models for stimulating innovation, having recently hit a 25-year low in the number of new drugs coming to market [37],[46].

## Rules of the System: Why We Need an R&D Treaty

The CEWG systematically assessed these various new approaches to improving the R&D system and found particularly promising open-access approaches that “de-link” the financing of R&D from the pricing of end products so that medicines can be sold near the cost of production. However, it also concluded that current efforts remain ad hoc, fragmented, and constrained by the tensions inherent in existing rules. Current initiatives lack a reliable, sustainable mechanism to generate sufficient funding for research, rely heavily on donor financing and priorities, and cover a limited set of diseases. Commenting on access to innovative medicines in developing countries, the head of Novartis succinctly said: “We have no model which would meet the need for new drugs in a sustainable way…. You can't expect for-profit organisations to do this in a large scale. If you want to establish a system where companies systematically invest in this kind of area you need a different system” [47].

An R&D treaty could complement and build on existing initiatives by addressing four areas that remain particularly weak: affordability, sustainable financing, efficiency, and equitable governance.

**1. Affordability:** Currently there is no system to ensure that new medicines will be affordable to the majority of people who need them. Despite major progress for HIV, no similar institutional arrangements exist for widely patented drugs in other therapeutic areas, such as the non-communicable diseases that account for nearly one-half of the burden of illness in LMICs [48],[49]. Affordability is likely to be an even bigger problem for biologics, for which generic competition is more limited than for traditional small-molecule drugs. A treaty could include measures to ensure affordability, including new incentive mechanisms that incorporate the principle of de-linkage. Examples include licensing through the Medicines Patent Pool or using treaty-generated funds to reward a prize to a successful drug developer, both of which allow for competitive generic production of the drug [27]. Such approaches could engender more equitable access to the benefits of scientific progress, but would also require reliable financial contributions across countries. Establishing the ground rules for such a system is likely to require a binding legal instrument.

**2. Sustainable Financing:** Currently there are no mechanisms to ensure sufficient, predictable financing of R&D to meet health needs in LMICs. Donor governments and non-profit entities, such as the Gates Foundation, have invested significantly in neglected disease R&D, with total global funding estimated at US$3 billion in 2010, and resulting in 140 products in development from a baseline of almost zero a decade ago [19]. While such progress is laudable, neglected disease R&D funding remains a small proportion of global spending (about 2%). And these figures cover only neglected diseases, not the broader range of R&D needs of neglected populations. However, at the same time that needs are increasing, for example as PDPs move promising compounds into clinical trials, neglected disease R&D funding has declined in the past year due to decreased donor contributions amidst the economic crisis [19]. This decline highlights the need for more sustainable financing arrangements, including the potential use of innovative financing mechanisms that tap into the growing capacity of middle-income countries to contribute to R&D. LMICs contributed an estimated US$5 billion to health R&D in 2005 [50].

A treaty could include binding obligations on governments to contribute to R&D, with due regard for varying ability to pay, thereby addressing the free-rider problem while establishing equitable burden-sharing arrangements. An international agreement is likely to be required to establish robust, sustainable, predictable, and sufficient financial flows for R&D.

**3. Efficiency in Innovation:** There is considerable room for improving the efficiency of the innovation process. For example, by impeding the free flow of information, intellectual property rights can retard the accumulation of common knowledge that drives forward scientific progress [51],[52]. Recognizing this problem, open-source R&D initiatives by publicly funded research labs or pre-competitive platforms among pharmaceutical firms have been established to facilitate knowledge-sharing; these initiatives hold promise, but remain few and nascent [53]. Another inefficiency arises from market incentives that reward the development of “me-too” drugs that are lucrative, but similar to pre-existing medicines and/or offer little or no therapeutic advance [54]. However, we have no global ground rules to facilitate open-source approaches, nor to counteract duplicative R&D investments in some areas or the relative neglect of others. A treaty could establish rules to improve efficiency in innovation. For example, global norms regarding research priorities and transparency in investment decisions could facilitate more efficient self-organization of the global scientific community. A treaty would not necessarily imply a centralized body directing all research activities, but rather, could establish rules that foster creativity such as incentives for faster global knowledge-sharing.

**4. Equitable Health-Focused Governance:** Market incentives, not health needs or public priorities, largely drive private R&D investments. A treaty could craft governance arrangements to ensure that the public interest drives innovation, rather than market-generated profits alone. For example, treaty rules could structure financial rewards for innovation so that they are commensurate with a medicine's health benefit [31]. In addition, as noted above, donors play a central role in financing the R&D now dedicated to the specific needs of developing countries—a welcome contribution, but one that also leaves priority-setting decisions largely in their hands. A system in which all countries contributed finances and knowledge could form the basis of more equitable governance arrangements in which affected populations have a stronger voice in decision-making.

Treaty proposals have included other measures, such as those to encourage regional cooperation among regulatory authorities, or enhance transparency in clinical trial results [55]–[58]. But since a comparative review of the proposals lies beyond the scope of this article, we have highlighted the four main systemic weaknesses above as forming the core rationale for a binding international instrument.

Some of these weaknesses can be addressed, at least in part, through national action. For example, patents can be licensed to improve affordability, and pharmacoeconomic assessments for medicines can link reimbursements to therapeutic efficacy. However, in a world in which information flows instantly across borders, research is carried out in dispersed networks, trade in medicines spans the globe, and intellectual property rules have already been globalized, coherent systemic change requires the negotiation of global rules. This, in turn, requires the collective engagement of governments.

The mere negotiation of a treaty will not be a panacea, and several areas require particular vigilance. First, as with most areas of international law, enforcement remains a thorny issue. Treaty negotiators will need to pay special attention to mechanisms that encourage compliance and manage free-riding. Such mechanisms could include traditional methods such as reporting and transparency requirements, as well as tools that have more teeth, such as provisions allowing countries that contribute *ex ante* to R&D through the treaty to pay lower prices for the medicines that are developed, while non-participating countries pay more. Second, treaty negotiations should not be seen as a replacement for ongoing policy experiments in new ways to generate innovation; such efforts should continue both for the immediate health benefits they can deliver through new products, and for the evidence they can provide to inform longer-term treaty design. Finally, treaty negotiations promise to be complex, lengthy, and resource-intensive. In order to merit such costly efforts, the final treaty must meet at least four key objectives: affordability of medicines, sustainable R&D finance, freer sharing of knowledge, and equitable governance that puts health at the system's core.

## Conclusions

Medical innovation and access to the fruits of scientific progress are no longer policy concerns restricted to the national level or to wealthy countries alone. In an era of health interdependence, effective tools for global governance are required to generate medical R&D as a global public good that can deliver benefits for all [11],[59]. A treaty is a promising tool for improving the coherence, fairness, efficiency, and sustainability of the global R&D system. It should be based on the understanding that a politically and financially sustainable system for generating health research will require both fair contributions from all, and fair benefit-sharing for all.

The recommendations of the CEWG can be seen as the product of nearly two decades of growing dissatisfaction with the shortcomings of the current R&D system. Leaders of governments, civil society, industry, and academia should seize this unprecedented opportunity to move forward.

## Supporting Information

Alternative Language Abstract S1 Translation of the Summary Points into Dutch (DOCX)

Alternative Language Abstract S2 Translation of the Summary Points into French and Spanish (DOC)

Alternative Language Abstract S3 Translation of the Summary Points into Portuguese (DOCX)

## Abbreviations

ARV: antiretroviral
CEWG: WHO Consultative Expert Working Group on R&D: Financing and Coordination
DNDi: Drugs for Neglected Diseases initiative
LMIC: low- and middle-income country
MMV: Medicines for Malaria Venture
PDP: product development partnership
R&D: research and development
TRIPS: Agreement on Trade-Related Aspects of Intellectual Property Rights
WHO: World Health Organization
WTO: World Trade Organization
