# Supplementary material for: Innovation and Access to Medicines for Neglected Populations: Could a Treaty Address a Broken Pharmaceutical R&D System?
Source: PLoS Med. 2012 May 15;9(5):e1001218. doi: 10.1371/journal.pmed.1001218 (PMC3352855; doi:10.1371/journal.pmed.1001218)
Supplement: Alternative Language Abstract S1 Translation of the Summary Points into Dutch — (DOCX) [file pmed.1001218.s001.docx]

Innovatie en de toegang tot geneesmiddelen voor genegeerde bevolkingsgroepen: Kan een internationaal verdrag een defect farmaceutische R & D-systeem helen?
Door Suerie Moon, Jorge Bermudez, en Ellen 't Hoen


Samenvatting:

• Het huidige systeem voor het onderzoek naar en ontwikkeling (R & D) van nieuwe geneesmiddelen voldoet onvoldoende aan de behoeften van de meerderheid van de wereldbevolking.
• Er is een gebrek aan nieuwe geneesmiddelen voor de "verwaarloosde ziekten", die voornamelijk bevolkingsgroepen raken met weinig koopkracht, en daarom onvoldoende stimulans bieden voor de industrie om te investeren in R & D. Echter, de R&D problemen gaan de smalle notie van ‘verwaarloosde ziekten’ ver te boven en het is daarom beter het probleem te benaderen vanuit de optiek van 'verwaarloosde bevolkingsgroepen".
• Internationaal debat en de voorstellen voor hervorming hebben geleid tot de aanbeveling dat nationale overheden onderhandelingen beginnen over een bindende medisch R & D verdrag om systematisch langdurige problemen met innovatie en mondiaal rechtvaardige toegang tot medicijnen aan te pakken. Ondanks de opkomst van tal van nieuwe initiatieven voor R & D die aan de behoeften van de ontwikkelingslanden voldoen, blijven de inspanningen tot nu toe ad hoc, versnipperd en ontoereikend.

• We bespreken hoe een R & D verdrag zou kunnen voortbouwen en aanvullen op bestaande initiatieven door het aanpakken van vier gebieden waar het systeem nog steeds bijzonder zwak is: betaalbaarheid, duurzame financiering, efficiëntie in innovatie, en billijk gezondheidscentraal bestuur.
 • We stellen dat effectieve instrumenten voor ‘global governance’ nodig zijn om medische R & D als een mondiaal publiek goed te genereren, gebaseerd op de verstandhouding dat een politiek en financieel duurzaam systeem zowel een reële bijdrage van allen als een eerlijke verdeling van de voordelen vereisen.
