# Supplementary material for: Innovation and Access to Medicines for Neglected Populations: Could a Treaty Address a Broken Pharmaceutical R&D System?
Source: PLoS Med. 2012 May 15;9(5):e1001218. doi: 10.1371/journal.pmed.1001218 (PMC3352855; doi:10.1371/journal.pmed.1001218)
Supplement: Alternative Language Abstract S2 Translation of the Summary Points into French and Spanish — (DOC) [file pmed.1001218.s002.doc]

**FRENCH translation**

**Points-clés :**

- Le système actuel de Recherche-Développement (R-D) ne répond pas convenablement aux besoins  de santé de la majorité de la population mondiale.
- Il y a un manque de nouveaux médicaments pour les « maladies négligées » - qui affectent en premier lieu les populations à faible revenu et ne représentent par conséquent pas un marché suffisamment attractif pour que l’industrie investisse en R-D. Toutefois, les problèmes vont bien au-delà de la seule notion de maladies négligées, et cette problématique se comprend donc mieux sous l’angle des « populations négligées ».
- Suite à plusieurs débats internationaux et propositions de réforme, il a été recommandé que les gouvernements démarrent des négociations sur un accord, de nature contraignante, portant sur la R-D médicale, qui permette de résoudre les problèmes récurrents d’innovation et d'accès équitable aux médicaments dans le monde.
- Malgré l'émergence de nombreuses nouvelles approches susceptibles de générer une R-D qui réponde aux besoins des populations les plus démunies, les efforts sont encore à ce jour ad hoc, dispersés et insuffisants.
- Dans cet article nous discutons comment un traité sur la R&D pourrait compléter et renforcer les initiatives existantes, notamment dans quatre domaines où le système actuel demeure encore particulièrement faible: l’accessibilité, la pérennité du financement, une innovation efficiente, ainsi qu’une gouvernance équitable centrée sur la santé.
- Dans cet article,  nous défendons la nécessité d’instruments efficaces de portée mondiale pour générer de la R-D médicale en tant que bien public mondial, étant entendu qu’un système politique et financièrement durable nécessite à la fois une répartition équitable des contributions autant que des bénéfices de la part de tous les acteurs.

SPANISH translation:

**Resumen de temas principales:**

-          El sistema actual de Investigación y Desarrollo (I+D) de nuevos medicamentos no cubre de manera adecuada las necesidades de la mayoría de la población mundial.

-          Hacen faltan nuevos medicamentos para las “enfermedades olvidadas” –aquellas que afectan principalmente a las poblaciones con bajo poder adquisitivo y que, por lo tanto, no representan un incentivo suficiente para que la industria invierta en I+D. Sin embargo, los problemas no se restringen a las llamadas enfermedades olvidadas; para comprender mejor la problemática se debe hablar de “poblaciones olvidadas”.

-          Se han llevado a cabo debates internacionales y propuestas de reforma, incluyendo la recomendación de que los gobiernos inicien las negociaciones para alcanzar un acuerdo vinculante sobre I+D médica, que permita atender problemas antiguos, sistemáticos, a través de la innovación y el acceso a medicamentos globalmente equitativo. A pesar de la aparición de numerosos nuevos enfoques capaces de generar una I+D que cubra las necesidades de las poblaciones más pobres, los esfuerzos siguen siendo *ad hoc*, fragmentados e insuficientes.

-          En este artículo se discute de qué manera un tratado sobre I+D podría complementar y construir sobre iniciativas existentes, centrándose en cuatro áreas donde el sistema sigue siendo particularmente débil: asequibilidad, sostenibilidad  financiera, eficiencia en la innovación, y una gobernabilidad equitativa centrada en la salud.

-          En este artículo, defendemos la necesidad de instrumentos para la gobernabilidad global, con el fin que se genere  una I+D médica como un bien público global, basado en el entendimiento de que un sistema política y financieramente sostenible, requiere tanto contribuciones justas por parte de todos, como un reparto de los beneficios equitativo.
