# Supplementary material for: Innovation and Access to Medicines for Neglected Populations: Could a Treaty Address a Broken Pharmaceutical R&D System?
Source: PLoS Med. 2012 May 15;9(5):e1001218. doi: 10.1371/journal.pmed.1001218 (PMC3352855; doi:10.1371/journal.pmed.1001218)
Supplement: Alternative Language Abstract S3 Translation of the Summary Points into Portuguese — (DOCX) [file pmed.1001218.s003.docx]

Innovation and Access to Medicines for Neglected Populations: Could a Treaty Address a Broken Pharmaceutical R&D System?

Suerie Moon1,2*, Jorge Bermudez3, Ellen ‘t Hoen4

1 Forum on Global Governance for Health, Harvard Global Health Institute, Cambridge, Massachusetts, United States of America,

2 Harvard School of Public Health, Boston, Massachusetts, United States of America,

3 Fundação Oswaldo Cruz, Rio de Janeiro, Brazil,

4 IS Academy HIV/AIDS, School for Social Science Research, University of Amsterdam, The Netherlands

Provenance: Commissioned; externally peer reviewed.

Resumo dos Pontos principais

· O Sistema atual para pesquisa e desenvolvimento (P&D) para novos medicamentos não responde adequadamente às necessidades da maior parte da população mundial.

· Existe uma carência de novos medicamentos para as “doenças negligenciadas” – Essas que afetam principalmente as populações com pouco poder aquisitivo, e consequentemente oferecem incentivos suficientes para que a indústria invista em P&D. No entanto, com uma problemática ultrapassando de longe a noção restrita de doenças negligenciadas, a questão é melhor compreendida pelo termo “populações negligenciadas”.

· O debate internacional e as propostas de reforma foram apresentadas, incluindo a que recomenda que os governos iniciem negociações a respeito de uma convenção para P&D médica vinculante para dar conta dos problemas sistemáticos e crónicos relacionados à inovação e ao acesso global equitativo a medicamentos. Apesar do aparecimento de várias novas abordagens para gerar P&D que responde às necessidades das populações mais pobres, esforços continuam ad hoc, fragmentados e insuficientes.

· Debatemos sobre como um tratado de P&D poderia complementar e reforçar iniciativas existentes ao enfatizar quatro áreas onde as iniciativas atuais são particularmente fracas: Preço acessível, financiamento sustentável, eficiência na inovação e governança baseada na saúde equitativa.

· Argumentamos que ferramentas efetivas para uma governança global são necessárias para gerar P&D na área médica que seja um bem publico global, baseado no entendimento de que um sistema politicamente e financeiramente sustentável vai requerer tanto contribuições equitativas de todos, como distribuição equitativa dos benefícios para todos.
